# Near infrared fluorescent imaging of choline kinase alpha expression and inhibition in breast tumors

**DOI:** 10.18632/oncotarget.14965

**Published:** 2017-02-01

**Authors:** Sean P. Arlauckas, Manoj Kumar, Anatoliy V. Popov, Harish Poptani, Edward J. Delikatny

**Affiliations:** ^1^ Department of Radiology, Perelman School of Medicine, University of Pennsylvania, Philadelphia, PA, United States; ^2^ Department of Cellular and Molecular Physiology, Institute of Regenerative Medicine, University of Liverpool, Liverpool, United Kingdom

**Keywords:** choline kinase, breast cancer, fluorescence optical imaging, chemotherapy

## Abstract

Choline kinase alpha (ChoKα) overexpression is associated with an aggressive tumor phenotype. ChoKα inhibitors induce apoptosis in tumors, however validation of their specificity is difficult *in vivo*. We report the use of optical imaging to assess ChoKα status in cells and *in vivo* using JAS239, a carbocyanine-based ChoKα inhibitor with inherent near infrared fluorescence. JAS239 attenuated choline phosphorylation and viability in a panel of human breast cancer cell lines. Antibody blockade prevented cellular retention of JAS239 indicating direct interaction with ChoKα independent of the choline transporters and catabolic choline pathways. In mice bearing orthotopic MCF7 breast xenografts, optical imaging with JAS239 distinguished tumors overexpressing ChoKα from their empty vector counterparts and delineated tumor margins. Pharmacological inhibition of ChoK by the established inhibitor MN58b led to a growth inhibition in 4175-Luc+ tumors that was accompanied by concomitant reduction in JAS239 uptake and decreased total choline metabolite levels as measured using magnetic resonance spectroscopy. At higher therapeutic doses, JAS239 was as effective as MN58b at arresting tumor growth and inducing apoptosis in MDA-MB-231 tumors, significantly reducing tumor choline below baseline levels without observable systemic toxicity. These data introduce a new method to monitor therapeutically effective inhibitors of choline metabolism in breast cancer using a small molecule companion diagnostic.

## INTRODUCTION

Choline kinase alpha (ChoKα) has become the subject of intense interest for its utility as an oncogenic biomarker as well as an anticancer therapeutic target [1–5]. ChoKα catalyzes choline phosphorylation as an intermediate step in the synthesis of two abundant membrane phospholipids, phosphatidylcholine (PtdCho) and sphingomyelin. Elevated ChoKα activity has been identified in approximately 40% of human breast tumors [6], and aberrant choline metabolism has been reported in lung, breast, colorectal, prostate, brain, bladder, and pancreatic cancers [7–10]. ChoKα produces phosphocholine (PC), which is critical for cell growth and for the regulation of cell proliferation by growth factors [11]. During malignant transformation, cells sequester high levels of PC that are detectable using ^1^H magnetic resonance spectroscopy (MRS) due to the nine chemically-equivalent protons on the methyl groups of the quaternary nitrogen [12,13]. ChoKα can be activated by the Ras and RhoA oncogenes, although overexpression of ChoKα alone is sufficient to transform cells to a malignant phenotype [14–16].

ChoKα inhibitors have been developed to study the involvement of this enzyme in malignant transformation. Shutting down *de novo* phospholipid biosynthesis leads to lower levels of pro-mitotic second messenger Kennedy pathway intermediates, increased ceramide levels, and de-stabilized endoplasmic reticulum [2, 17–19]. The most potent of these agents, TCD-717, is a promising anti-cancer drug [19] that is being evaluated in clinical trials [20]. MN58b, a *bis*-cationic ChoKα inhibitor, has been demonstrated to be lethal to lymphoma cells, but causes a reversible cell cycle arrest in normal cells [17]. Although the mechanism by which ChoKα inhibition causes cell death may involve scaffolding in addition to enzymatic functions [21], measurement of total choline-containing metabolite (tCho) levels by MRS is still the primary method of validating ChoK inhibitors *in vivo* [22, 23]. A limitation of this approach is that metabolite levels are also affected by the contributions of phospholipases, organic cation transporters, and sphingomyelinases [3, 24, 25]. Moreover, cell death can lead to deceptive decreases in tCho in MR spectra, requiring the measurement of secondary biomarkers [26, 27]. ^18^F and ^11^C choline PET tracers are useful for identifying ChoK inhibition [28], but choline tracer accumulation can be affected by choline transport inhibitors [29, 30] which have known toxicities [31]. In addition, recent reports have shown that ChoKα protein scaffolding, rather than the enzymatic function, may be critical for supporting cell viability [21, 32, 33]. Miyake and Parsons reported a c-Src-dependent link between ChoKα and EGFR [32]. More recent studies showed that small molecule non- symmetric ChoKα inhibitors with low nM IC_50_s could substantially reduce the metabolic product PC but only cause reversible growth arrest with no effects on cell viability [21, 33]. Thus further development of fluorescence-based imaging strategies that report on enzyme expression rather than enzyme activity is needed.

We have recently reported fluorescent small molecule choline mimetics (JAS239) that effectively attenuate choline phosphorylation. The structural similarity between symmetric, bis-heterocyclic ChoKα inhibitors and a class of carbocyanine dyes used for optical imaging led to the development of these enzyme inhibitors with near infrared fluorescence (NIRF) [4]. Within this wavelength range human tissue is relatively transparent [34–36] and NIRF optical imaging probes can be detected through several millimeters, and up to centimeters of tissue [37, 38]. Moreover, these probes exhibit a concentration dependent cellular uptake that cannot be attenuated in the presence of excess free choline indicating that they enter the cell independently of the choline transporters [4]. There is a particular need for more specific agents to assist surgeons in distinguishing tumor from normal tissue [36, 39], and intraoperative imaging is an expanding field for which NIRF offers an inexpensive and effective method of delineating tumor margin and assessing lymph node involvement [40–42]. In this work we investigate JAS239 as a NIRF ChoKα-targeted optical imaging probe in murine orthotopic breast tumors and compare this diagnostic method to MRS. *In vitro*, JAS239 was validated against MN58b and shown to have comparable potency to block cell growth and inhibit choline phosphorylation in breast cancer cell lines. *In vivo*, we demonstrate that JAS239 can be used to distinguish overexpression of ChoKα in breast tumor models and monitor ChoKα inhibition induced by MN58b. At higher doses, JAS239 was compared to MN58b to assess the therapeutic potential of ChoKα inhibitors in these tumor models. A therapeutic course of JAS239 was able to significantly reduce tumor tCho levels, reduce tumor cell density, decrease proliferation, and cause apoptosis and tumor growth arrest.

## RESULTS

### Characterization of inhibitor sensitivity in a breast cancer cell line panel

Inhibition of ChoKα by JAS239 and MN58b was measured in 4 breast cancer cell lines, MCF7 overexpressing ChoK (MCF7-CK+) [43], the corresponding empty vector cell line (MCF7-EV), MDA-MB-231, and 4175-Luc+, an MDA-MB-231 derivative harvested from a lung metastasis and transfected with luciferase [44]. The MCF7-CK+ line was confirmed to have elevated ChoKα expression and activity relative to MCF7-EV cells (Supporting Information, Supplementary Figure 1A–1B) [4]. No significant difference in ChoKα expression or activity was observed between the triple-negative MDA-MB-231 and 4175-Luc+ lines (Supplementary Figure 1A–1B). MN58b attenuated the elevated ChoKα activity in MCF7-CK+ cells with similar efficacy to the control MCF7-EV cell line as indicated by the IC_50_ (Table 1, Supplementary Figure 1C). Similarly, no significant difference in IC_50_ was found between the MDA-MB-231 and the 4175-Luc+ cell lines after 2 h of MN58b treatment (Table 1, Supplementary Figure 1D). MN58b was at least twice as effective as JAS239 at reducing choline phosphorylation at 2 h in all cell lines tested (Table 1, Supplementary Figure 1C–1F).

**Table 1:** Potency of MN58b and JAS239 in a panel of breast cancer cell lines

	IC_50_ (μM)		EC_50_ (μM)	
Cell Line	MN58b	JAS239	MN58b	JAS239
MCF7-EV	3.85 ± 0.42	11.1 ± 5.64	3.29 ± 0.48	4.04 ± 0.55
MCF7-CK+	2.36 ± 0.28	10.3 ± 2.48	6.86 ± 0.43	5.33 ± 0.84
MDA-MB-231	4.36 ± 1.37	9.92 ± 0.22	14.26 ± 4.6	27.2 ± 7.9
4175-Luc+	4.59 ± 0.55	9.89 ± 0.24	20.7 ± 6.4	35.0 ± 21.6

Cells were treated with varying concentrations of MN58b or JAS239 and analyzed after 2 h for ^14^C-PC production or after 17 h for cell viability. Data for 3 separate experiments were fitted to sigmoidal plots and IC_50_s and EC_50_s are reported ± SEM for the radiotracing and viability assays, respectively.

Extended treatment of cancer cells with ChoK inhibitors leads to cell death by apoptosis [19]. After 17 h, we found no significant difference in the EC_50_s between MN58b (Table 1, Supplementary Figure 2A) and JAS239 in the MCF-7 EV and CK+ cell lines (Table 1, Supplementary Figure 2B) by Trypan blue exclusion. As was found in radiotracing assays, MN58b was more potent at inhibiting cell growth than JAS239 in MDA-MB-231 and 4175-Luc+ cells although the results were more variable (Table 1, Supplementary Figure 2C–2D).

### JAS239 colocalization with ChoKα

**Figure 1:**
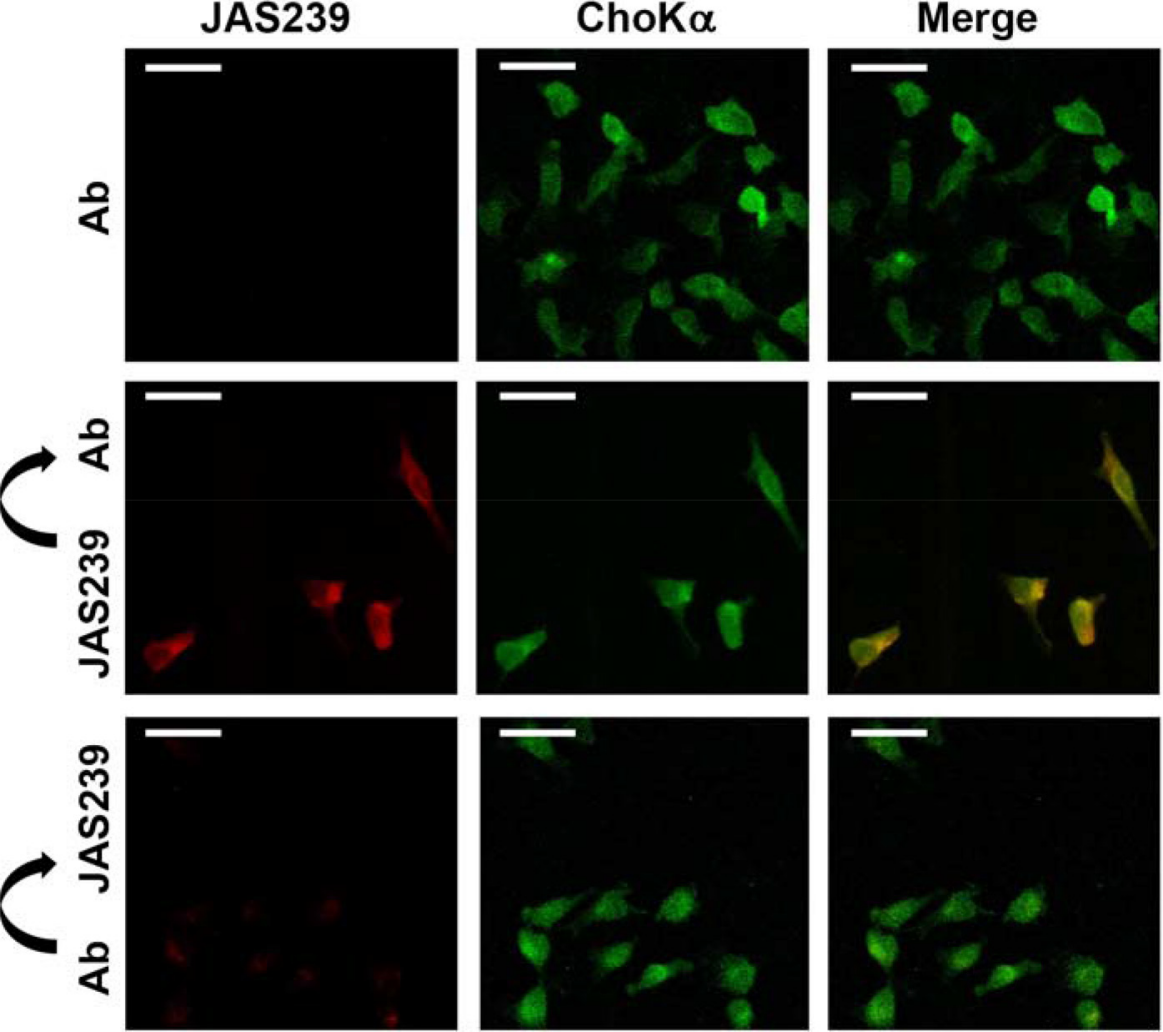
JAS239 colocalizes with ChoKα Fixed permeabilized MDA-MB-231 cells treated with a ChoKα-specific antibody (Ab) followed by a Texas Red-conjugated secondary antibody (Ex. 543 nm; Em. 565–615 nm) were assessed by confocal microscopy (top row). In cells stained with JAS239 followed by the ChoKα antibody (middle row), strong colocalization between the JAS239 (Ex. 633 nm; Em. > 650 nm) and ChoKα is observed. Cells incubated with ChoKα antibody prior to JAS239 staining (bottom row) no longer retain the NIRF probe. Scale bar represents 35 μm.

Fluorescence confocal micrographs of fixed MDA-MB-231 cells treated with an anti-human ChoKα antibody are shown in Figure 1, top. When treated individually, no overlap was observed between the Texas-Red (ChoKα antibody) and JAS239 channels (see also Supplementary Figure 3A). MDA-MB-231 cells stained with JAS239 and probed with the ChoKα antibody revealed strong colocalization (Figure 1, middle and Supplementary Figure 3A). Of the ChoKα-positive pixels, 89 + 9% were also positive for JAS239 (Supplementary Figure 3B). Similarly, 89 + 10% of JAS239-positive pixels were positive for ChoKα (Supplementary Figure 3C). When cells were pre-treated with ChoKα antibody, marked reduction in JAS239 retention was detected (Figure 1, bottom). Reduced JAS239 signal caused a lower Pearson's Correlation Coefficient, although 84 + 4% of the regions which were ChoKα-positive were also JAS239-positive (see Supplementary Figure 3B), and 87 + 2% of the JAS239-positive pixels were ChoKα-positive (see Supplementary Figure 3C). This demonstrates that JAS239 retained in these cells was still associated with ChoKα, even though the intensities followed a different distribution trend. These results are consistent with JAS239 being out-competed by the antibody for binding sites.

### NIRF imaging facilitates study of JAS239 biodistribution

**Figure 2:**
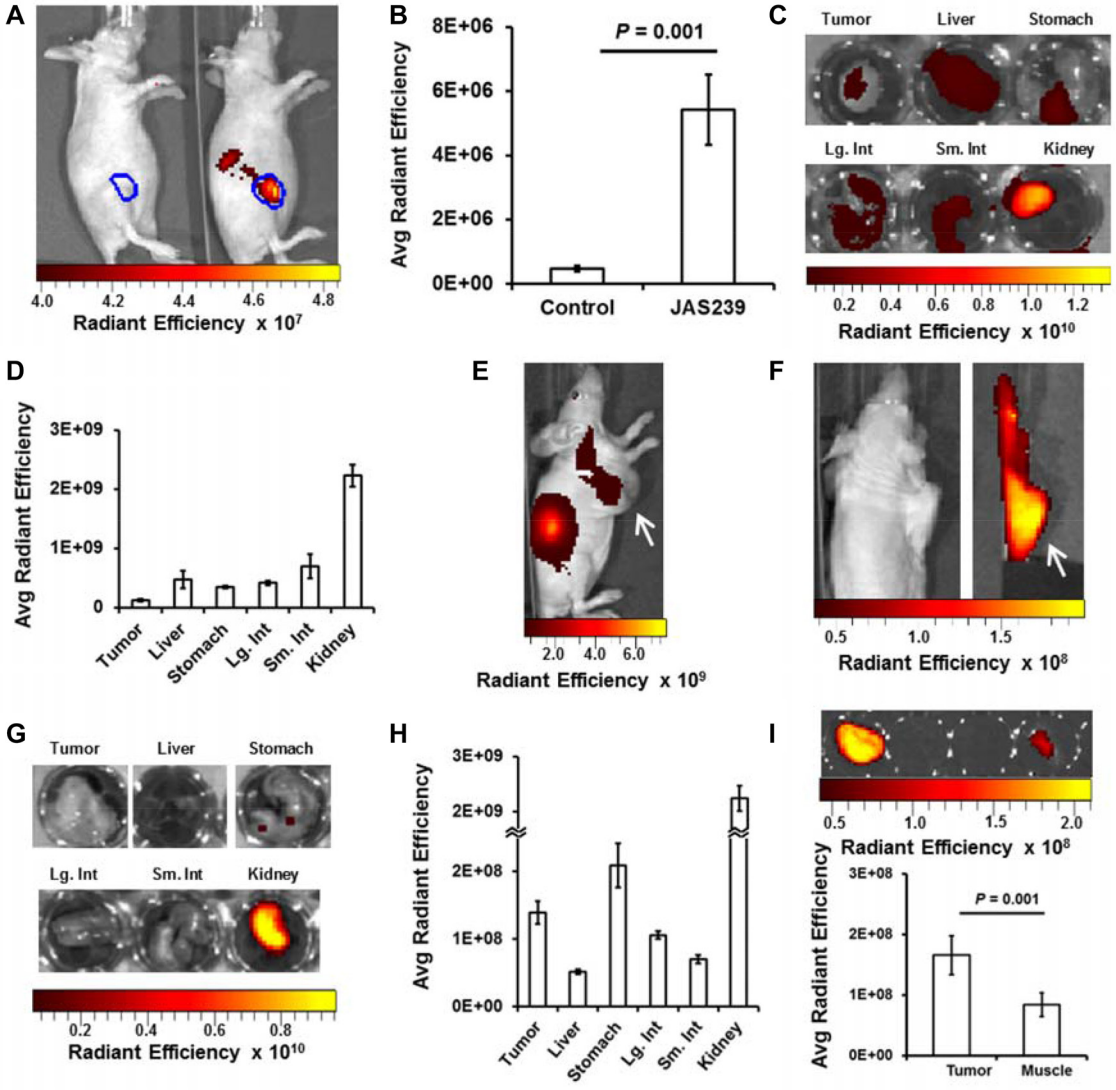
*In vivo* optical imaging provides JAS239 biodistribution data (**A**) Tumor margin (blue) defined by bioluminescent imaging of 4175-Luc+ tumors shows no NIRF in vehicle-injected mouse (left) but intratumoral NIRF in right mouse injected with JAS239 in Tween-80/Tris Buffer. Renal excretion of JAS239 is also seen outside the tumor boundaries. (**B**) Quantified Average Radiant Efficiency [p/sec/cm^2^/sr]/[μW/cm^2^] in tumor ROIs (*n* = 4). (**C**, **D**) Organs resected 90 min following JAS239 injection imaged for NIRF (C) and quantified for comparison. (E) Delivered in an ethanol/saline formulation, maximal intratumoral JAS239 (white arrow) relative to background was achieved 24 h post-injection. (**E**, **F**) Tumors (white arrow) moved to shoulder allow organs involved in JAS239 excretion to be cloaked. (**G**, **H**) Resected organs 24 h post-injection of JAS239 imaged for NIRF (G) and quantified (H). (**I**) Tumor and muscle NIRF were quantified and were found to be significantly different. Unless otherwise noted, cohort size was 5 animals and values represent ± SEM. Total JAS239 delivered was 20 nmol/animal.

Tumors derived from 4175-Luc+ cell lines had the most rapid *in vivo* growth (see Supplementary Figure 4A–4B) that could additionally be monitored with bioluminescence imaging. Bioluminescence in mice bearing orthotopic 4175-Luc+ tumors was measured 15 min following luciferin injection (Supplementary Figure 5A). This signal was used to delineate the tumor margins (in blue) and was used to confirm that the bioluminescence did not overlap with the NIR range (Supplementary Figure 5B). The next day no residual luminescence was detected, and mice were treated with control vehicle (Figure 2A, left mouse) or 20 nmol JAS239 in Tween-80/Tris buffer (Figure 2A, right mouse). After initial hepatic clearance (approximately 75 min), mice were injected i.p. with luciferin and imaged for bioluminescence and NIRF 15 min later. Bioluminescence was again used to delineate the tumor margin (Supplementary Figure 5C), and no NIRF signal was detected in the control animals (Figure 2A, left mouse; *n* = 5). In the JAS239-injected animals, NIRF was emitted both from the tumor and from the kidneys (Figure 2A, right mouse; *n* = 4). NIRF emission at 800 nm was an order of magnitude stronger in JAS239-injected animals vs. control (Figure 2B). The maximum NIRF contrast between the tumor and background was achieved 90 minutes post-JAS239 injection, tumor fluorescence diminished after this time due to excretion (Supplementary Figure 5D).

Mice from this cohort were injected one week later (allowing the first dose to clear entirely) with JAS239, euthanized 90 min later, and the organs resected and imaged (Figure 2C). The biodistribution of JAS239 is consistent with the renal signal detected in whole animals, however the signal from other organs (liver in particular) was higher than would be expected from whole-body images (Figure 2D). The tumor contrast seen in whole-body NIRF images is enhanced due to the relative depth of these organs, compared to the surface tumor.

A cohort of 5 mice was inoculated with subcutaneous 4175-Luc+ tumors high on the right flank. This cohort was injected with JAS239 in an ethanol/saline vehicle. Fluorescence from the kidneys was the dominant source of NIRF signal (Figure 2E). Intentional placement of these tumors on the shoulders allowed us to cloak the lower extremities and isolate the tumor for NIRF imaging (Figure 2F). Tumor signal to noise was highest at 24 h and the organs in these animals were resected and imaged to estimate biodistribution (Figure 2G). Compared to the Tween-80/Tris vehicle, hepatic signal from JAS239 in this formulation dropped nearly one order of magnitude, drastically reducing background signal (Figure 2H). At this time, a nearly two-fold tumor-to-muscle ratio was found *ex vivo* (Figure 2I).

### Optical imaging of JAS239 to assess ChoKα status

**Figure 3:**
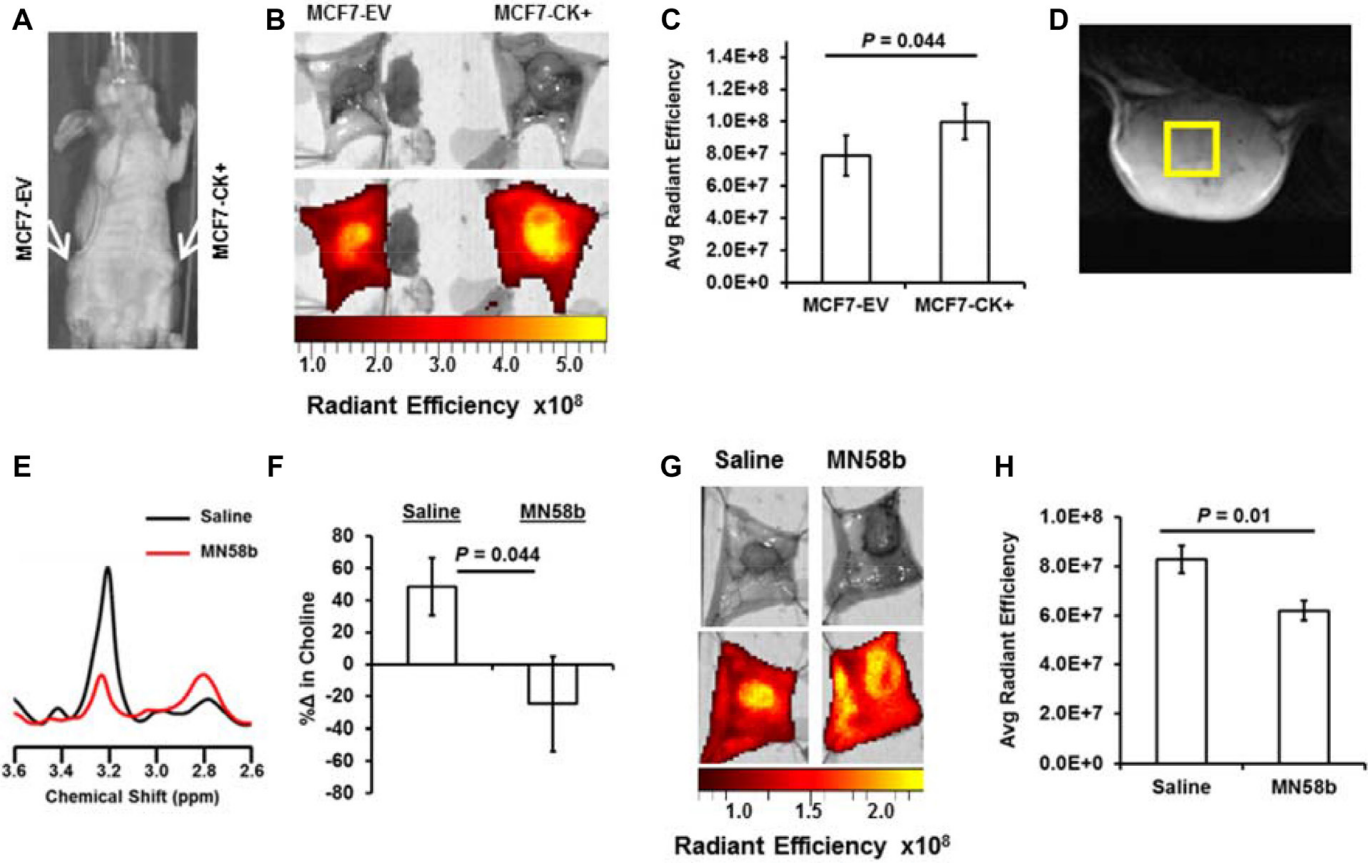
NIRF imaging for JAS239 accumulation can detect genetic overexpression and pharmacologic inhibition of ChoKα (**A**) Athymic nude mice were inoculated with MCF7-EV (left mammary fat pad) and MCF7-CK+ (right mammary fat pad) cells. (**B**) Tumor-bearing fat pads were surgically exposed and imaged for NIRF 24 h after JAS239 injection in an ethanol formulation. (**C**) Quantified Average Radiant Efficiency [p/sec/cm^2^/sr]/[μW/cm^2^] of resected fat pads reveals enhanced JAS239 retention in ChoKα-overexpressing tumors (*n* = 7). (**D**) Representative *T*_2_-weighted MR image of a 4175-Luc+ tumor used to plan 3×3x3 mm^3^ voxel placement. (**E**) *In vivo* MR spectra of the choline (3.2 ppm) and PUFA (2.8 ppm) regions for control (black) and MN58b-treated (red) tumors. (**F**) MN58b-treatment prevents the increase in intratumoral tCho (3.2 ppm) observed between scans in the control cohort. (**G**, **H**) NIRF of the exposed saline (left) or MN58b-treated (right) tumors (G) was quantified and MN58b-induced ChoKα inhibition diminishes JAS239 retention significantly (H). For saline and MN58b groups, *n* = 5. Values are reported as ± SEM.

The effect of genetic overexpression of ChoKα on JAS239 retention was studied in mice bearing orthotopic MCF7-EV and MCF7-CK+ tumors (Figure 3A) that were injected with JAS239 in ethanol/saline vehicle. Optical imaging of the surgically exposed mammary fat pads revealed distinct NIRF signal within the confines of the orthotopic tumor, and tumor margins could clearly be delineated (Figure 3B). JAS239 accumulation was significantly higher in MCF7-CK+ tumors compared to contralateral MCF7-EV tumors (Figure 3C). A significant but more moderate increase in NIRF was observed between exposed MCF7-EV and MCF7-CK+ fat pad tumors in mice injected with JAS239 in Tween-80/Tris buffer 90 min prior (see Supplementary Figure 5E).

MN58b treatment was used to model pharmacologic inhibition of ChoKα. Mice bearing orthotopic 4175-Luc+ tumors were imaged with *in vivo* MRS and then treated with a 5-day dose regimen of MN58b at 2 mg/kg/day i.p. *In vivo* MRS 7 days later (Figure 3D–3E) showed reduced tCho levels after MN58b treatment (Figure 3E–3F). In control-treated animals tCho levels increased substantially during the one-week period (Figure 3F). These animals underwent post-treatment JAS239 injections with follow-up NIRF imaging (Figure 3G), and MN58b treatment reduced intratumoral JAS239 accumulation relative to untreated tumors (Figure 3H).

### JAS239 is a therapeutically effective ChoKα inhibitor in breast xenograft models

**Figure 4:**
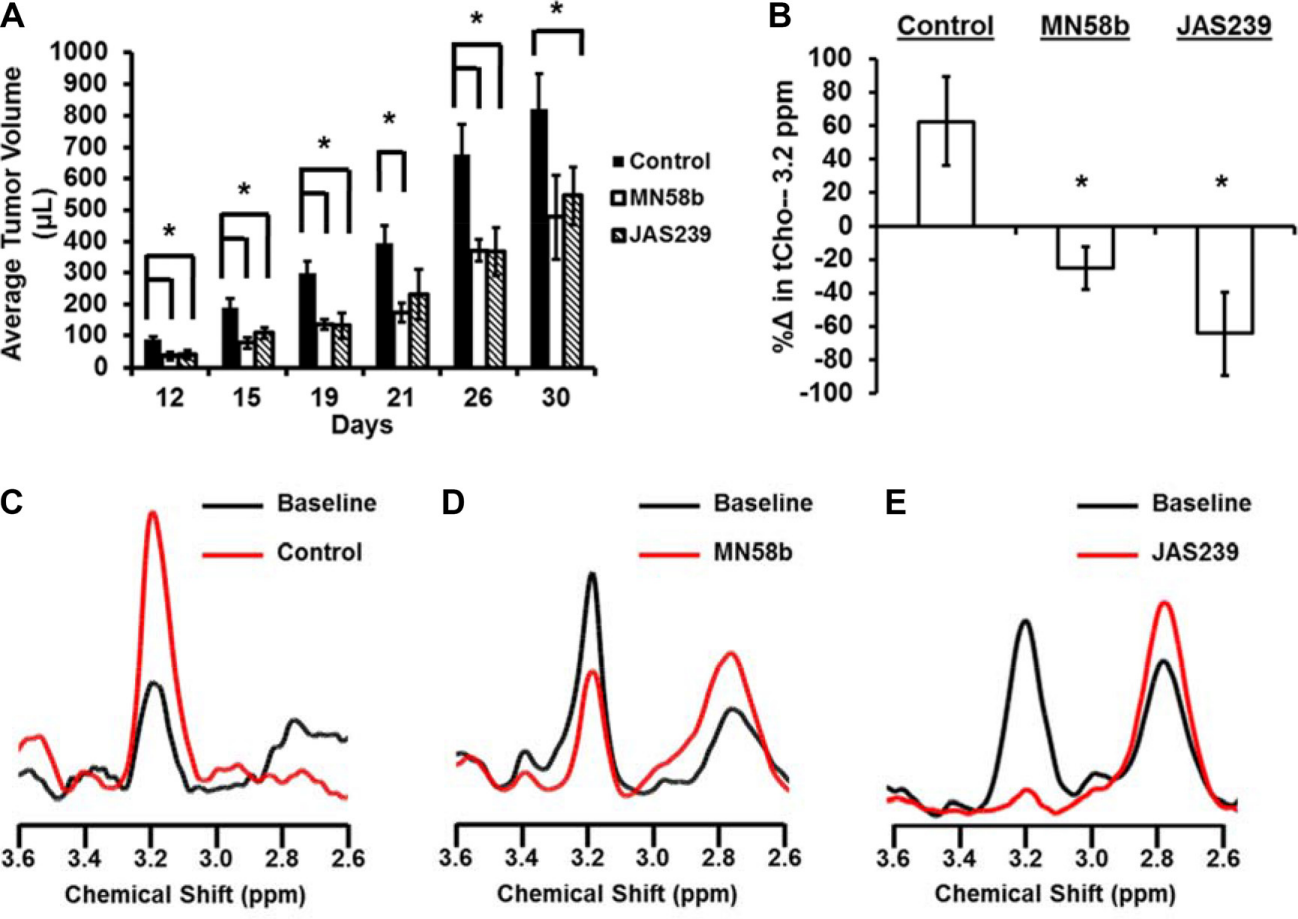
JAS239 exhibits ChoKα inhibition and slows breast tumor growth (**A**) Treatment for 5 consecutive days with 2 mg/kg of MN58b (*n* = 4) or 4 mg/kg of JAS239 (*n* = 5) significantly reduced the growth rate of orthotopically-implanted 4175-Luc+ tumors, compared to control animals (*n* = 4). (**B**–**E**) The change in tCho (3.2 ppm) within MDA-MB-231 tumors was measured using MRS (B) to show progressive tCho increase during saline treatment (*n* = 5) (B, C), whereas reductions in tCho in response to 5 days of 2 mg/kg MN58b (*n* = 4) (B, D) or 4 mg/kg JAS239 (*n* = 3) (B, E) were observed. Values are reported as ± SEM, *indicates *P* < 0.05 compared to the control group.

To test the therapeutic potential of JAS239, a group of athymic nude mice with 4175-Luc+ tumors were given a 5-day i.p. dose treatment regimen of JAS239 or MN58b beginning 3 days after orthotopic tumor inoculation. Both 2 mg/kg/day MN58b-treatment and 4 mg/kg/day JAS239-treatment resulted in significantly smaller tumor volumes compared to control animals (Figure 4A). Until day 26, both the MN58b and JAS239 cohorts had significantly smaller (*P* = 0.021 and 0.022, respectively) tumors than the vehicle-treated group.

To study the metabolic effects of JAS239 therapy on tumors, tCho was used as a pharmacodynamic marker of ChoKα inhibition (Figure 4B). MDA-MB-231 xenografts were used because prior characterization of MN58b in this model [22] allowed for the confident use of this inhibitor as a positive control. MRI volume measurements indicated that vehicle-treated tumors grew during the week between pre- and post-treatment MRS scans (Paired *t-test*: *P* = 0.009), whereas MN58b-treated and JAS239-treated tumors were static during the same time span (Supplementary Figure 6A). Single voxel MRS in each tumor (Supplementary Figure 6B–6D), showed that tCho increased during the time course in controls (Figure 4B–4C). Significant reductions in tCho compared to control were observed in response to MN58b (*P* = 0.006, Figure 4B, 4D) and JAS239 (*P* = 0.004, Figure 4B, 4E). JAS239 treatment was able to reduce tCho to noise levels in these tumors. Polyunsaturated fatty acid (PUFA) resonances, a measure of tumor apoptosis [45], increased after MN58b and JAS239 treatment, although this trend was not significant (Supplementary Figure 6E). Supplementary Figure 6F depicts a summary of the toxicology of the mice from this experiment, revealing no significant effects except for elevation of blood urea nitrogen in MN58b-treated animals compared to controls (*P* = 0.012).

**Figure 5:**
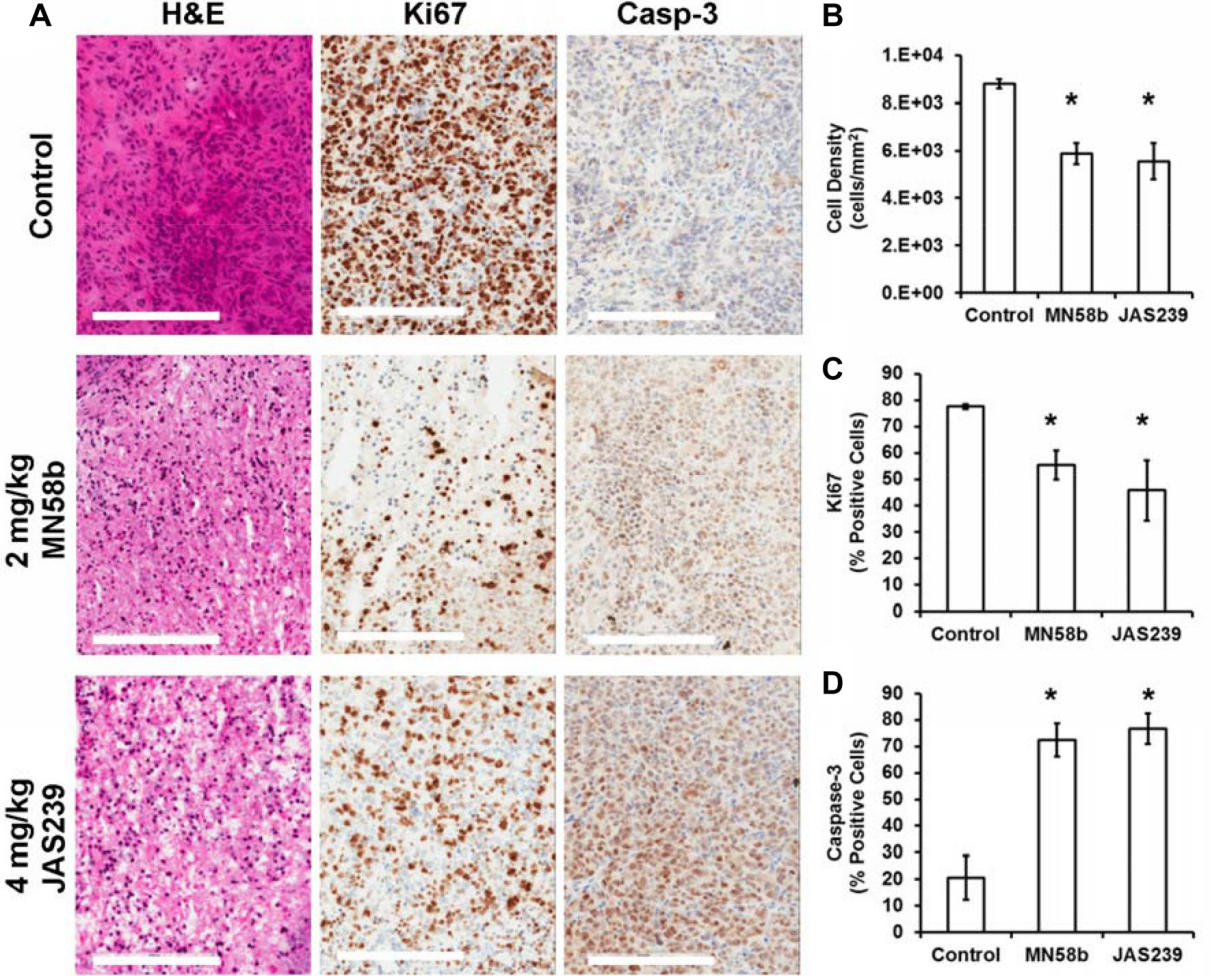
Histological assessment of tumors reveals reduced cell density, lower proliferation, and elevated apoptosis in response to ChoKα inhibitors (**A**) H&E staining (left column) of MDA-MB-231 tumor xenografts reveals lower cell density in MN58b (middle row) and JAS239 (bottom row) treated tumors compared to vehicle-treated tumors (top row). Reduced Ki67-positive nuclei (middle column) and heightened caspase-3 positive cells (right column) were also found in MN58b and JAS239 treated tumors. Significant (**B**) reduction in cell density, (**C**) lower Ki67 positivity, and (**D**) elevated caspase-3 were observed in both MN58b and JAS239 treated tumors relative to control, indicating apoptosis in response to ChoK inhibition. Scale bars represent 200 μm. Values are reported as ± SEM and *n* = 3 for each treatment cohort. *indicates *P* < 0.05.

Tumors from MDA-MB-231-bearing mice were harvested for histological assessment immediately after follow-up MRI/MRS scanning (Figure 5A). Evaluation of H&E slides (Figure 5B) showed that both MN58b (*P* = 0.007) and JAS239 (*P* = 0.01) caused significant reduction in cell density. MN58b (*P* = 0.026) and JAS239 (*P* = 0.034) treatment significantly reduced the percentage of Ki67-positive cells compared to the control group (Figure 5C). Consistent with reduced cell number and proliferation, higher caspase-3 levels (Figure 5D) were detected in MN58b (*P* = 0.016) and JAS239 (*P* = 0.015) treated tumors than in controls.

## DISCUSSION

JAS239 is a ChoKα inhibitor whose inherent near infrared fluorescence makes it possible to measure its interaction with cancer cells. In this study, we investigated the ability of JAS239 to detect ChoKα expression in human xenograft models of breast cancer. We first verified the response to ChoKα inhibition by JAS239 in a panel of human breast cancer cells representing different germ lines (epithelial and mesenchymal), tumor stages (estrogen-positive and triple-negative), derivation (mouse-naïve and mouse-adapted), and ChoKα expression (low and high). Using confocal microscopy, JAS239 staining was compared with ChoKα expression measured using immunohistochemistry. Colocalization of the probe with ChoKα was observed, indicating a direct interaction that could be blocked when the N-terminal antibody was applied first. This is consistent with a mechanism of competitive inhibition of the active site of ChoKα [4].

Against pure yeast ChoKα, the average reported IC_50_s of the bis-pyridinium and bis- quinolinium ChoKα inhibitors are 37.1 and 33.9 μM, respectively; the antiproliferative activities, EC_50_s, in HT-29 cells are 19.7 and 3.7 μM, respectively [46–47]. Although determined in different systems, the measured IC_50_ of 4.6 μM for JAS239 represents potency comparable to or better than 85% of the reported bis-pyridinium–based ChoKα inhibitors, and 61% of the bis-quinolinium compounds. The EC_50_ values are also comparable, although determined using different assays in cell lines of different tissue origin. The action of JAS239 against MDA-MB-231 cells suggests ChoKα inhibition may be an effective strategy in triple- negative breast cancers, which are often therapy resistant. Its potency suggests that further modifications based upon the carbocyanine template, and subsequent structure–activity characterization, may yield compounds with higher specificity to ChoKα.

While *in vitro* data can often be promising, translation to *in vivo* models is crucial for a true assessment of the efficacy and feasibility of this technology. *In vivo* biodistribution showed that JAS239, a cationic small molecule, accumulated and was cleared through the kidneys. An initial flush was observed in the liver where ChoKα is naturally abundant [48], but this is also the site of first-pass metabolism. With the guidance of bioluminescence imaging in 4175-Luc+ tumor xenografts, intratumoral JAS239 NIRF was detectable but eventually drowned out by renal filtration and excretion of the probe. This result does not disparage the translational utility of this probe, as clinical optical imaging is not a whole-body but rather a tissue or region-specific measurement, especially when employed in an intraoperative setting [39].

While biodistribution studies showed that JAS239 could be detected in other major organs, the tumor/muscle ratio suggests that measurements confined to the breast region were robust enough to distinguish malignant from normal tissue. This was further verified when tumor-bearing mammary fat pads of JAS239-injected mice were surgically exposed and imaged with NIRF optical imaging. JAS239 accumulation was found to be a reliable identifier of ChoKα overexpressing tumors in matched MCF7-EV and CK+ tumors. Although the difference in JAS239 fluorescence between EV and CK+ tumors was moderate, the observed tumor/normal tissue fluorescence ratio of 2.8 ± 0.2 was sufficient to distinguish malignant from normal tissue and is similar to values reported for other agents currently being used for fluorescence-guided tumor resection [42]. To assess the validity of this imaging strategy we employed the well-studied ChoKα inhibitor MN58b as a positive control [17, 19, 49] and evaluated the effects of MN58b therapy in tumor-bearing mice with NIRF imaging and MRS. The parallel use of two imaging techniques indicated a more complete story: MN58b bound to tumor ChoKα (blocking JAS239 accumulation) thus attenuating flux through the Kennedy pathway (lowering tCho signals). It is important to note that the 25 + 30% reduction in tCho measured by *in vivo* MRS was mirrored by the 25 + 8% reduction in JAS239 accumulation, but NIRF measurements were less variable between animals resulting in greater statistical confidence. Moreover, optical imaging was easier to perform. While MRS required 1 h per mouse — including set-up, anesthesia, shimming, and scanning — the optical imaging technique could be performed on up to 5 mice simultaneously and required a simpler procedure involving one i.v. injection and follow-up surgery 24 h later.

To explore the therapeutic properties of JAS239, its ability to inhibit tumor growth and attenuate tCho in murine breast xenografts was determined. In orthotopic breast tumors, JAS239 significantly reduced tumor growth rate to the same degree as MN58b. MN58b at 4 mg/kg had previously been reported to lower PC levels in MDA-MB-231 tumor xenografts [22]. No observable or measurable toxicity was found using 4 mg/kg JAS239, and 2 mg/kg MN58b appeared tolerable but post-mortem toxicological studies revealed evidence of elevated blood urea nitrogen indicative of impaired renal function in these mice. Prior reports indicated MN58b to be tolerable when dosed daily for 5 days at 5 mg/kg, but lethal at 10 mg/kg [49]. An optimized bis-pyridinium ChoKα inhibitor was recently shown effective at 10 mg/kg daily for 3 days, and body weight measurements indicated no adverse toxicities [50]. Both JAS239 and MN58b were capable of significantly reducing intratumoral tCho and increasing PUFA resonances associated with apoptosis [23, 45]. However, JAS239 was more effective at reducing tumor tCho, decreasing the levels down to baseline noise. In control tumors, rising tCho measured by *in vivo* MRS was an indicator of tumor growth during the week between pre- and post-therapy. Tumor status was also evaluated by histological methods. Both ChoK inhibitors effectively reduced cell number, reducing the proliferation marker Ki67 and inducing widespread cell death via apoptosis.

Expanding interest in ChoKα inhibitors for cancer therapy has raised the need for rapid and reliable methods to validate these therapies *in vivo* [5]. There have been concerns with the reliability of RECIST measurement as a marker of treatment responsiveness [51]. An addendum to the first clinical trial of TCD-717 included MRS evaluation of tCho levels as a supplemental treatment response indicator (Clinical Trials identifier: NCT01215864). MRS is the current gold standard for choline measurement *in vivo* due to its specificity, non-invasive acquisition, and feasible inclusion following MRI for RECIST measurements. To understand the degree to which choline metabolites represent a real biomarker of tumor progression and response to therapy, it is crucial to develop methods of identifying the oncogenes that contribute to heightened choline in cancerous tissue. ChoKα has been reported throughout the literature as a key contributor to aberrant choline metabolism [52], and its regulative role in cell growth and division directly links the Kennedy pathway to cancer malignancy. The use of NIRF choline mimetics to determine ChoKα status in whole tumors will provide a measure of histological tumor grade, a potential indicator of tumor margins during surgical resection, and a validation method for evaluating ChoKα inhibitors in the clinic.

## MATERIALS AND METHODS

Cell lines: MDA-MB-231 human breast cancer cells were obtained from the ATCC and authorized via COI assay, STR analysis, and BacT/ALERT 3D. The 4175-Luc+ cell line, transfected to express luciferase and green fluorescent protein and derived from a MDA-MB-231 lung metastasis [44], was acquired from Dr. Andy Minn and maintained in 5 μg/mL blasticidin (Invitrogen), which was not included during experiments. Both cell lines were maintained in DMEM (Mediatech). MCF7 breast cancer cells (*Chk-4* clone, MCF7 CK+) or the empty vector (MCF7 EV) were provided by Drs. Zaver Bhujwalla and Tariq Shah [43] and cultured in MEM (Mediatech) supplemented with 400 mg/mL G418 sulfate (Mediatech) that was not included during experiments. All cells were supplemented with 10% FBS (HyClone Laboratories), 1% penicillin/streptomycin (Mediatech), and 1% L-glutamine (Mediatech), and kept in a 37^°^C humidified atmosphere (5% CO_2_). Cell lines were tested bi-monthly for mycoplasma. Cells were frozen in liquid nitrogen and used only at low passage numbers. Each cell line was characterized for ChoKα expression by Western blot [4].

### Cell viability assays

MCF7 cells were plated for Trypan exclusion assays at 1.0 × 10^6^ cells/well in 6-well plates and treated with MN58b or JAS239, synthesized in house [4]. At 17 h, cells were collected by trypsinization, stained with Trypan Blue, and counted using a Neubauer hemocytometer. For 3-(4,5-Dimethylthiazol-2-yl)-2,5-diphenyltetrazolium bromide (MTT) assays, cells in 96-well plates (7.5 × 10^4^ cells/well) were treated with JAS239 or MN58b overnight, and stained with 20 μL of 5 mg/mL MTT (Sigma-Aldrich) for 2 h, lysed with DMSO and scanned for absorbance using a SpectraMax M5 plate reader (Molecular Devices) at 550 nm, subtracting background absorbance at 690 nm from each sample.

### Chokα activity assay

Cells in 6-well plates (1.0 × 10^6^ cells/well) were incubated for 24 h then treated with JAS239 or MN58b. After 1 h, cells were spiked for 1 h with 0.5 μCi/mL [methyl-^14^C]-choline (PerkinElmer) and fixed with trichloroacetic acid. Aqueous cellular extracts were separated by thin layer chromatography (TLC) and the ^14^C-PC production measured by autoradiography on a Fujifilm FLA-7000 using previously described methods [4, 23].

### Confocal microscopy

MDA-MB-231 cells plated on sterile glass coverslips were fixed in 4% paraformaldehyde, permeabilized in 0.2% Triton X-100, and blocked in 1% BSA. Staining with 0 or 200 μM JAS239 was performed 30 min prior or 1 h after application of rabbit anti-human ChoKα antibody (ab38290; Abcam, 1:1000 dilution). Coverslips were then stained with goat anti-rabbit antibody conjugated to Texas Red (Vector Laboratories; TI-1000, 1:500), dehydrated, and mounted with VectaShield (Vector Laboratories). Confocal micrographs were acquired on a Zeiss LSM 510META NLC using excitation (Ex.) 543 nm and emission (Em.) 565–615 nm for Texas Red and Ex. 633 nm and Em. > 650 nm for JAS239. The Coloc 2 FIJI software plug-in was used to assess colocalization of the anti-ChoKα antibody with JAS239 by calculating Pearson's Correlation Coefficients and Manders’ Colocalization Coefficients for at least 3 separate ROIs [53].

### Animal model and tumor cell implantation

All animal studies were approved by the Institutional Animal Care and Use Committee of the University of Pennsylvania. Six week old female athymic nude Foxn1^nu/nu^ mice (National Cancer Institute) were housed in a temperature-controlled environment with 12 h light/dark cycle. Anesthesia was induced using 2% isoflurane in oxygen for all experiments. For MCF7 tumors, mice were supplemented with 60-day release 0.36 mg 17β-estradiol tablets (Innovative Research of America) using a trochar in the nape of the neck. After 1 week, 4 × 10^6^ cells in Matrigel (BD Biosciences) were inoculated into the left (CK+) and contralateral (EV) mammary fat pads. Subcutaneous xenografts, including those analyzed in MRI/MRS studies, were grown on the shoulder to allow for cloaking of the kidneys during optical imaging studies. Tumor growth was determined via caliper measurement of three orthogonal dimensions and volume calculated as length * width * height * π/6.

### Optical imaging

Bioluminescence and NIRF (Ex. 745 nm; Em. 800 nm) images were acquired in an IVIS Spectrum (PerkinElmer). NIRF is measured in Average Radiant Efficiency, photons per second (p/sec) corrected for the area of the region of interest (ROI) and the efficiency of the lamp (μ Watt *cm^−2^ / steradian^-1^). For bioluminescence imaging, mice were injected i.p. with 150 mg/kg of firefly *D*-luciferin (Biosynth). Bioluminescent Radiance (p/sec/cm^2^/sr) was monitored by time-course imaging until the luminescence intensity plateaued. For NIRF imaging, mice were injected i.v. with 100 μL vehicle or JAS239 (20 nmol). Two vehicle formulations were tested in mice bearing 4175-Luc+ tumors: the first was 0.1% Tween-80 / 50 mM Tris-HCl, and the second was 1% ethanol diluted in 0.9% saline. ROIs with a 20% threshold were created from the bioluminescence image using the LivingImage software (PerkinElmer) program, and this region was used to analyze intratumoral JAS239 accumulation in the NIRF images. Resected organs were placed in 24-well plates and NIRF was measured to quantify JAS239 biodistribution. For diagnostic studies, mice bearing both MCF7-EV and MCF7-CK+ tumors of approximately 200 μL were injected with 20 nmol JAS239 in Tween-80/Tris (8 mice) or in ethanol/saline (7 mice). The cohorts were euthanized after 90 min / 24h respectively, and tumor-bearing flanks were surgically exposed. NIRF from the tumor-bearing fat pads was subtracted from background muscle signal and the Average Radiant Efficiency measured.

### *In vivo* MRI

#### Animal preparation

A mouse was anesthetized and secured to a custom-built restraining device to minimize motion artifacts and the head positioned within a nose cone delivering isoflurane. A respiration pillow was placed dorsally to monitor respiration rate, and a thermister was inserted rectally to measure body temperature. Both sensors were connected to a small animal monitoring device (SA Instruments). The tumor was placed into a slotted-tube resonator (inner diameter = 11 mm, depth = 9 mm) built in-house and the probe was centered in the magnet. Body temperature was regulated at 37 ± 1°C by blowing warm air into the magnet via a thermostatically controlled device (SA Instruments).

#### *In vivo* single voxel spectroscopy

Mice bearing subcutaneous 4175-Luc+ or MB-MDA-231 tumors (∼300 μL) underwent pre-treatment MRS scans in a 9.4 T horizontal bore magnet equipped with 40 G/cm gradients interfaced to an Agilent Direct-Drive console (Agilent) operating vnmrj 2.3.C software. Multi-slice gradient and spin echo images were used for tumor localization. *T*_2_-weighted spin echo anatomical images were acquired using a spin echo multi-slice (SEMS) pulse sequence (TR = 1000 ms, TE = 10 ms, number of slices = 20, field of view = 20 × 20 mm^2^, slice thickness = 1 mm, NT = 1, matrix size = 256 × 128) and used for voxel selection. A single voxel ^1^H PRESS spectrum was acquired in a 3×3x3 mm^3^ voxel (TR = 3000 ms, TE_1_ = 12.68 ms and TE_2_ = 10.01 ms, number of averages = 128, complex points = 4096, spectral width = 4000 Hz and acquisition time of 6 min 24 sec). Water suppression was achieved using the VAPOR sequence [54]. An unsuppressed water spectrum was acquired (8 averages) as a chemical shift and concentration reference. One week following initiation of treatment, ^1^H MR spectra were acquired again. Spectra were processed using Mnova Lite 5.2.5 software (Mestrelab Research). Free induction decays were processed by 10 Hz apodization followed by Fourier transformation. Spectra were phased and chemical shift and baseline correction performed prior to fitting of the water (4.7 ppm), tCho (3.2 ppm), PUFA (2.8 ppm), and lipid (1.3 ppm) resonances. The metabolite/water ratio was determined and the percent change was determined as [post-treatment ratio – pre-treatment ratio] *100%/pre-treatment ratio.

### Animal treatments

#### Companion diagnostic study

*In vivo* MRS was used to establish baseline tCho levels in a cohort of 10 mice bearing 4175-Luc+ tumors. Mice were injected i.p. for 5 consecutive days with either 100 μL saline or 2 mg/kg MN58b. A follow-up MR scan was acquired 1 week after treatment. After the final MRS, each animal was injected i.v. with a trace dose (20 nmol) of JAS239 in ethanol/saline. After 24 h, the mice were euthanized and each tumor was analyzed for NIRF as described above.

#### Tumor growth inhibition

A cohort of 15 mice was inoculated with 4175-Luc+ tumors. Beginning 3 days post-inoculation, animals received a 100-μL i.p. injection of either control DMSO/saline, 2 mg/kg MN58b or 4 mg/kg JAS239 in DMSO/saline daily for 5 days. Tumor volumes were assessed by caliper measurement.

#### Pharmacodynamic indicators of Chokα inhibition

A cohort of 15 mice was inoculated with MDA-MB-231 cells. When a volume of 300 μL was reached, tumors were scanned using MRS. Tumor volumes were measured using *T*_2_-weighted images. The animals were separated into treatment groups, and for 5 consecutive days each group was injected i.p. with 100 μL of DMSO/saline vehicle (*n* = 5), 2 mg/kg MN58b (*n* = 4), or 4 mg/kg JAS239 (*n* = 3). Initial cohort sizes were 5 per condition, but some animals had to be removed from the study because the treatment was so effective that we were unable to place voxels within post-treatment tumors that were too small. Follow-up MRI/MRS scanning was performed on day 7 to assess tumor growth inhibition and changes in metabolite levels. After imaging, blood was collected into heparin-coated tubes by cardiac puncture. Resected tumors were embedded in Optimum Cryo-Temperature (Sakura Finetek), flash-frozen in liquid nitrogen, and stored at –80^°^C. Blood samples were submitted to the Ryan Veterinary Clinic Diagnostic labs for toxicology.

### Histopathology

Hematoxylin and eosin staining and immunohistochemistry was performed as described previously [23]. Caspase-3 (R&D Systems AF835) and Ki67 (Abcam ab16667) antibody staining was performed on a Bond Max automated staining system (Leica Microsystems) using the Bond Intense R staining kit (Leica Microsystems DS9263). An Aperio Scan Scope OS (Aperio Technology) was used to scan slides, and the Image Scope nuclear staining algorithm (version 9; Aperio Technology) was used to quantify Ki67 and caspase-3 positive nuclei in representative regions of each slide.

### Statistical analysis

Statistical analyses of treated versus control values were performed in Microsoft Excel 2010 (Version 14.0.7128.5000 32-bit) using an unpaired Student's *t-test* unless noted otherwise. A *P-value* of ≤ 0.05 was considered to be statistically significant. All error bars represent mean ± SEM.

## SUPPLEMENTARY FIGURES


