# Supplementary material for: Near infrared fluorescent imaging of choline kinase alpha expression and inhibition in breast tumors
Source: Oncotarget. 2017 Feb 1;8(10):16518–30. doi: 10.18632/oncotarget.14965 (PMC5369982; doi:10.18632/oncotarget.14965)
Supplement: Supplementary file 1 [file oncotarget-08-16518-s001.pdf]

Near infrared fluorescent imaging of choline kinase alpha expression and inhibition in breast tumors

Supplementary Materials

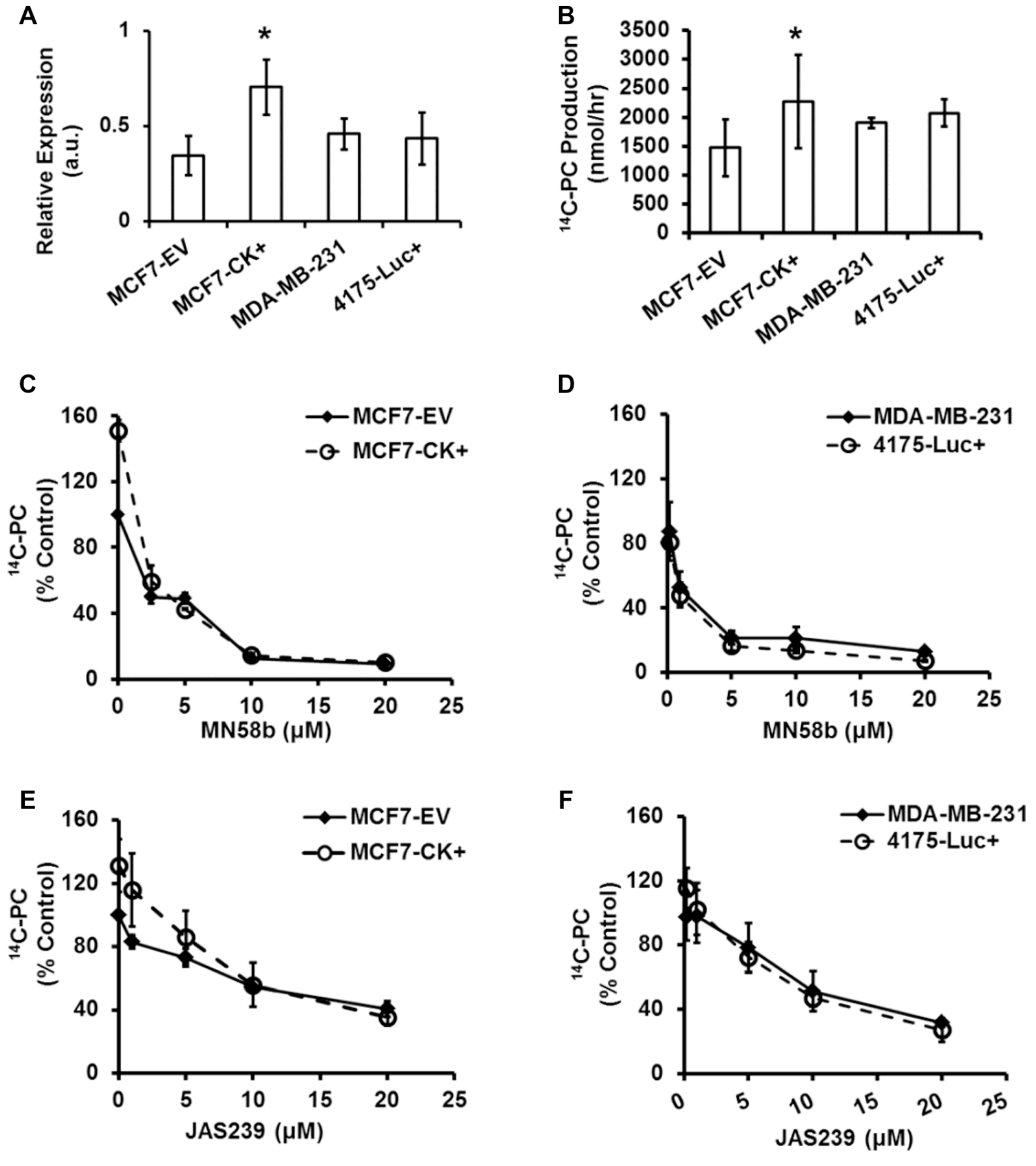

Supplementary Figure 1: Characterization of ChoKα in a panel of human breast cancer cell lines. (**A**) Determination of ChoKα expression by Western blot confirms elevated ChoKα protein found in MCF7-CK+ cells. (**B**) Conversion of ^{14}C-choline to ^{14}C-PC follows the pattern expected from ChoKα expression levels in (A). (C–F) Dose-response curves comparing the effect of MN58b (**C, D**) or JAS239 (**E, F**) on ^{14}C-choline phosphorylation in MCF7-EV and MCF7-CK+ cells (C, E) or MDA-MB-231 and 4175-Luc+ cells (D, F). Values are reported as a percentage of the untreated EV group (for MCF7 cells) or untreated MDA-MB-231 group (for MDA-MB-231 and 4175-Luc+), and represent ± SEM for 3 separate experiments.

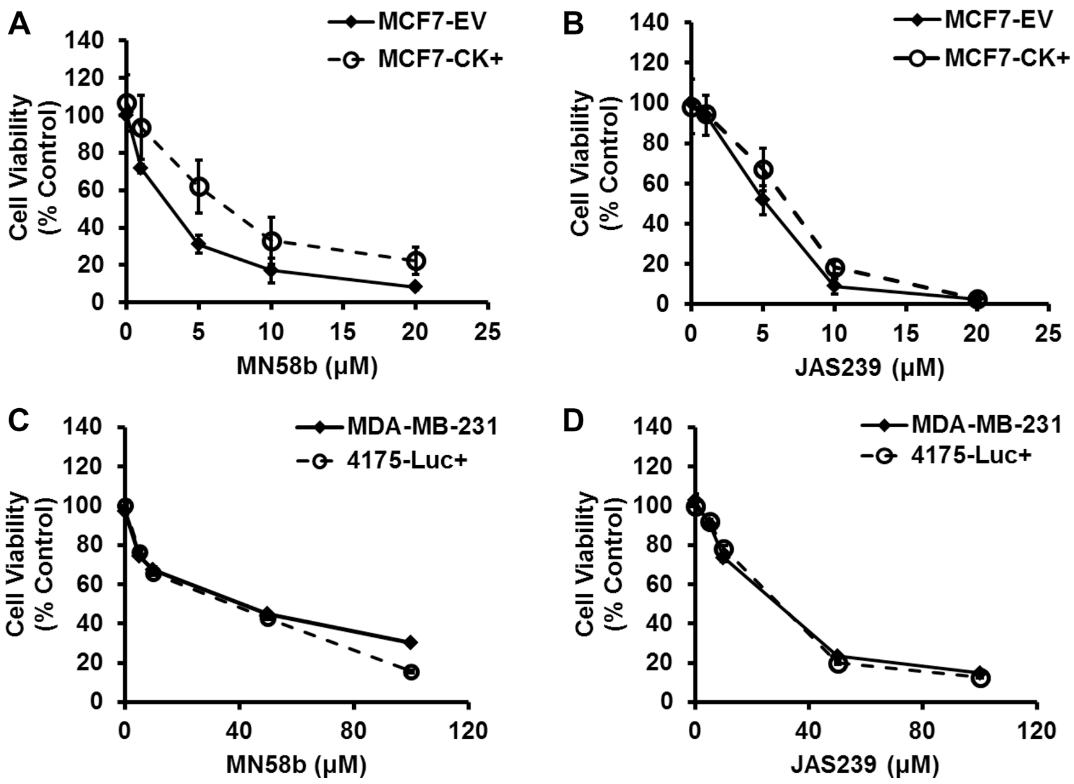

Supplementary Figure 2: Breast cancer cell viability in response to ChoK inhibitors. (**A–D**) Cell viability measurements via Trypan blue exclusion demonstrate comparable cytotoxicity in MCF7-EV and MCF7-CK+ cells (A, B) exposed overnight to either MN58b (A) or JAS239 (B). MTT assays were performed on MDA-MB-231 and 4175-Luc+ (C, D) cells exposed to MN58b (C) or JAS239 (D). Values are reported relative to untreated control groups and represent ± SEM for 3 separate experiments. At higher JAS239 concentrations, the MTT assay was used as JAS239 interfered with Trypan blue counting. There was no overlap in absorbance readings between the MTT formazan product and JAS239.

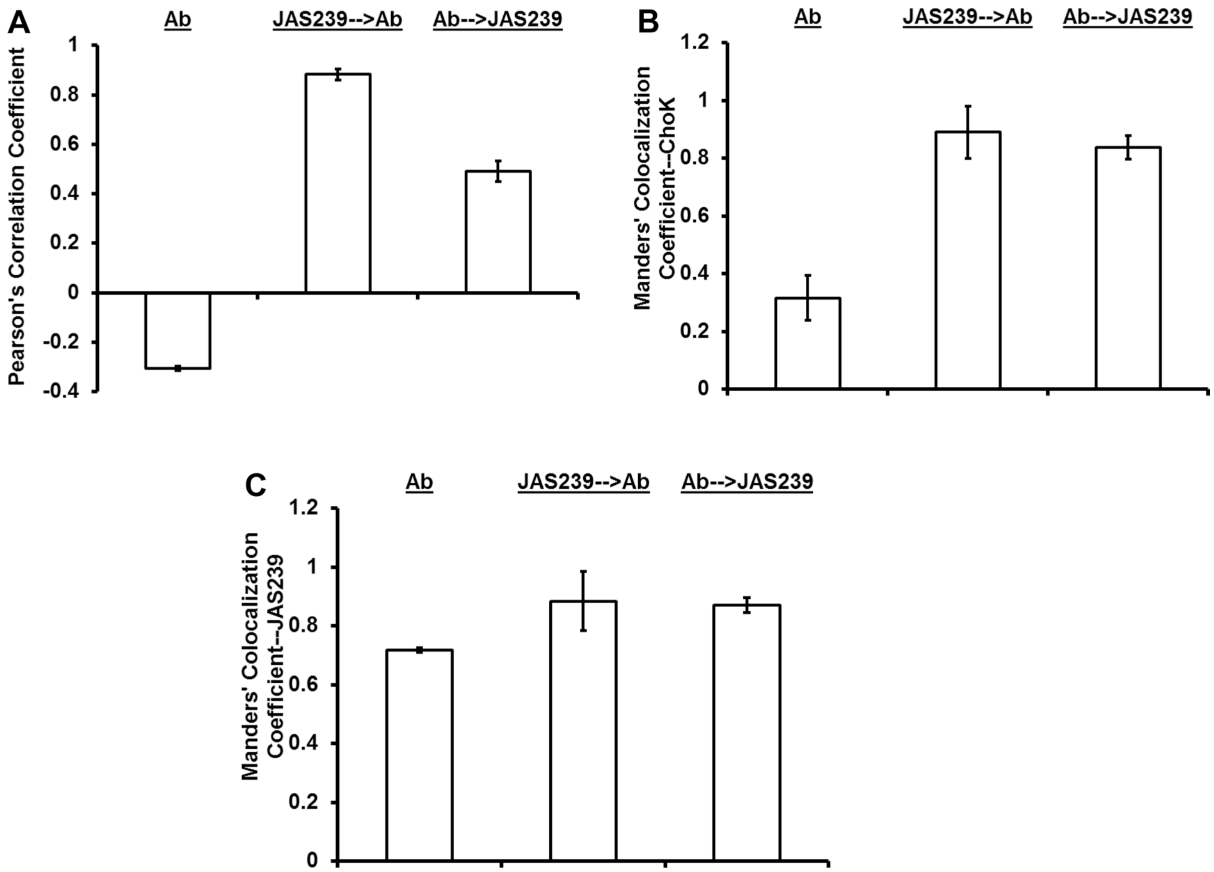

Supplementary Figure 3: JAS239 exhibits strong colocalization with ChoKα in MDA-MB-231 cells. (**A**) JAS239 is strongly correlated with ChoKα, unless fixed cells are pre-incubated with ChoKα antibody (Ab). (**B**) Manders' Colocalization Coefficients calculated for the Texas-Red channel confirm that the majority of ChoKα is bound by JAS239 when JAS239 is present. (**C**) Manders' Colocalization Coefficients calculated for the JAS239 channel indicate that the majority of JAS239 that is present intracellularly is co-distributed with ChoKα. Values represent ± SEM for 3 separate fields of view.

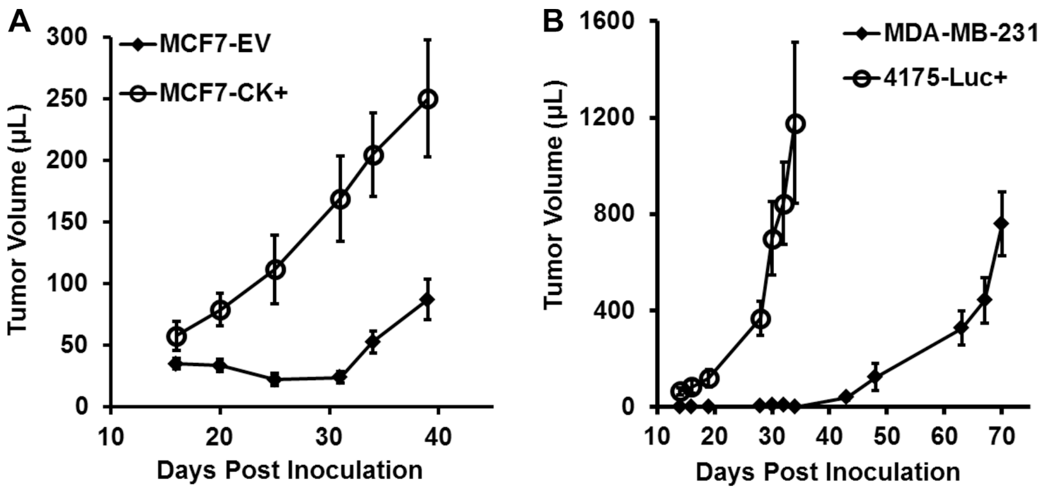

Supplementary Figure 4: Tumor growth patterns in the panel of human breast cancer derived cell lines. (**A**) ChoKα overexpression accelerates tumor growth in an MCF7-derived orthotopic xenograft model. (**B**) Orthotopically inoculated 4175-Luc+ derived tumors exhibit accelerated growth in volume compared to orthotopic MDA-MB-231 xenografts. Values represent ± SEM for 5 animals per cohort.

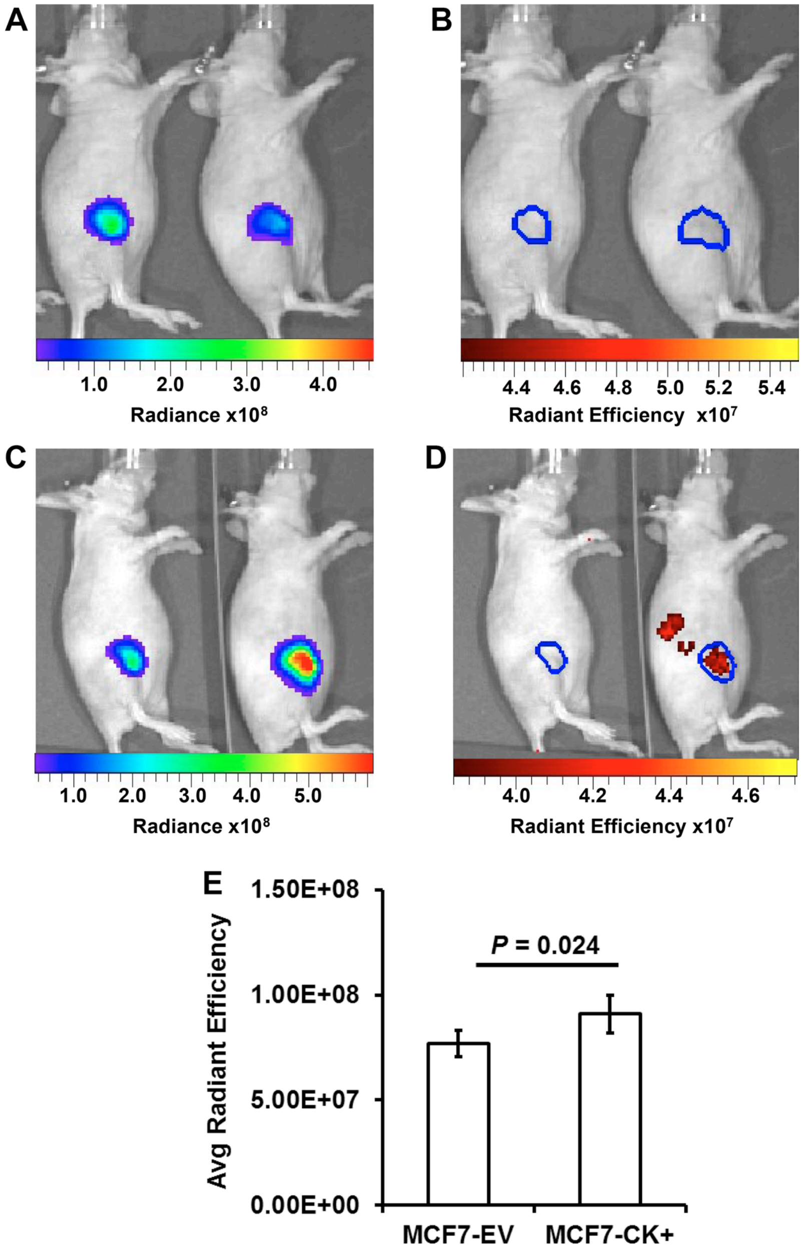

Supplementary Figure 5: Bioluminescence of 4175-Luc+ tumors delineates tumor margin for NIRF measurement and ChoK measurement. (**A**) Two representative 4175-Luc+ tumor-bearing mice imaged for bioluminescent Radiance [p/sec/cm^2/sr] 15 min after injection of luciferin. (**B**) The photons emitted from the bioluminescence reaction were used to draw an ROI (blue line) around the tumor margin, and was confirmed to be undetectable in the near infrared filter range. (**C**) Mice injected with either control vehicle (left) or JAS239 were administered luciferin after 75 min and imaged 15 min later for bioluminescence to define tumor ROIs. Heightened radiance was observed in JAS239 mice presumably due to absorption of photons by JAS239 and emission at a longer, more tissue transparent wavelength. (**D**) The tumor NIRF (right mouse) begins to diminish relative to the renal signal 95 minutes after JAS239 injection. (**E**) Quantified Average Radiant Efficiency [p/sec/cm^2/sr]/[μW/cm^2] of fat pads bearing MCF7-EV or MCF7-CK+ tumors show significantly increased fluorescence 90 min after injection of 20 nmol JAS239 in a Tween-80/Tris buffer. Values represent ± SEM for 8 animals.

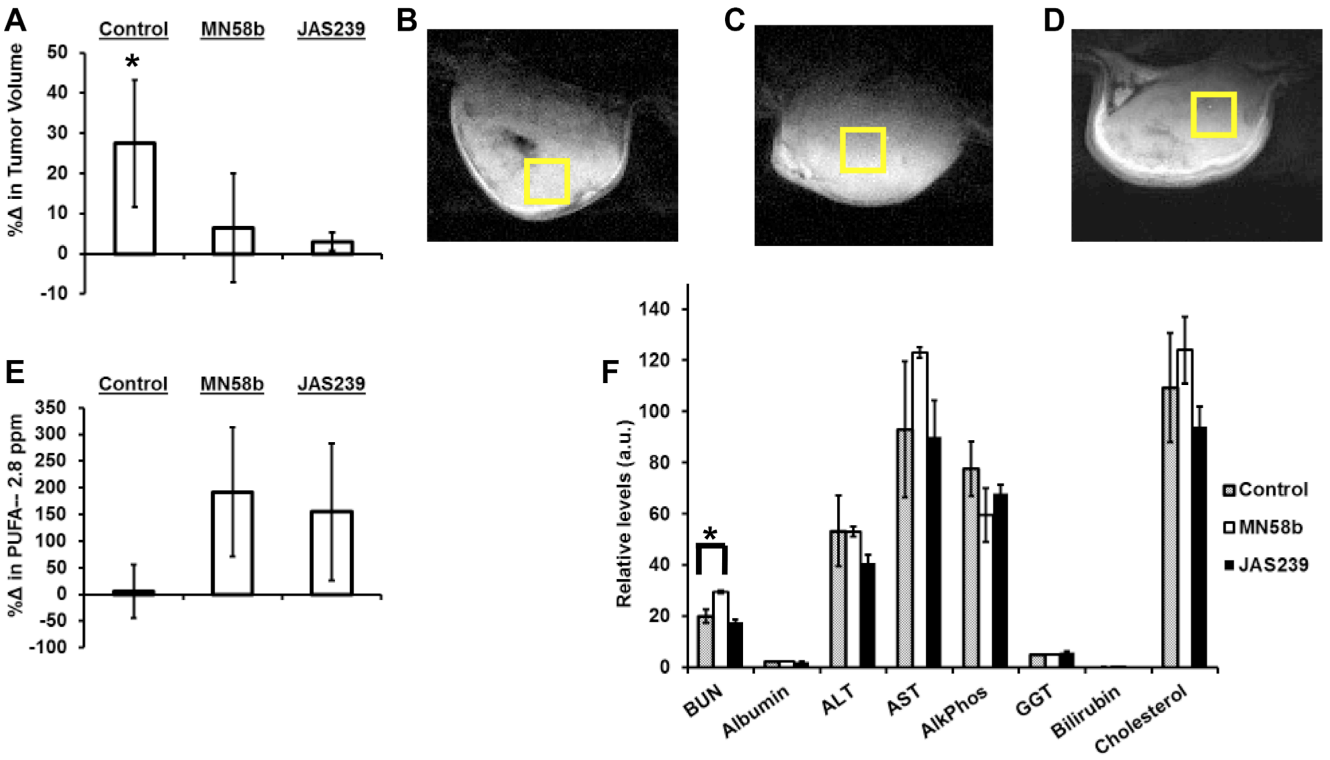

Supplementary Figure 6: Both ChoKα inhibitors show early signs of tumor growth inhibition and apoptosis, however JAS239 is better tolerated in animals. (**A**) Vehicle-treated tumors increased in size during the treatment regimen, whereas the tumor volumes of MN58b and JAS239 treated mice were unchanged. (**B–D**) T_2-weighted MRI images of MDA-MB-231 tumors used for volume measurements in (A) and placement of 3 × 3 × 3 mm^3 voxels. (**E**) PUFA resonances at 2.8 ppm are unchanged in the control cohort and increase in response to treatment with both MN58b and JAS239, but these changes are not statistically significant. (**F**) A profile of enzyme function and blood metabolite levels reveals that MN58b-treated animals had elevated blood urea nitrogen (BUN) relative to control animals. No signs of elevated albumin, alanine transaminase (ALT), aspartate transaminase (AST), alkaline phosphatase (AlkPhos), gamma-glutamyl transferase (GGT), bilirubin, or cholesterol changes were found in response to either MN58b or JAS239 treatment. Three animals were included per cohort and values represent ± SEM. * indicates $P < 0.05$.
